# EMERGING MR IMAGING AND SPECTROSCOPY APPLICATIONS FOR AGING RESEARCH IN MODEL ORGANISMS

**DOI:** 10.1093/geroni/igad104.1276

**Published:** 2023-12-21

**Authors:** Rafael de Cabo, Mustapha Bouhrara

## Abstract

Advances in quantitative magnetic resonance imaging and spectroscopy (MRI/MRS) methodology have enabled probing tissue microstructure and function with exquisite specificity and sensitivity in human and model systems. Using this technology to examine changes in tissue microstructure and function in aging or pathology has the potential to provide a window into the underlying age-related diseases’ mechanisms, and to nominate MR biomarkers for longitudinal assessment and intervention. Water molecules within biological tissues undergo interactions with their environment through nuclear relaxation, magnetization transfer, chemical exchange, and diffusion. These processes are sensitive to underlying local tissue properties such as density, microstructure, temperature, acidity, composition, and geometry. Through use of combinations of magnetic fields and radiofrequency pulse formalisms, MRI/MRS provides unique sensitivity and specificity to probe these mechanisms in all biological tissues. Advances in acquisition strategies, hardware designs, computational analyses, and signal modeling have positioned MRI/MRS as powerful emerging noninvasive modalities to studying biological tissue to differentiate normal from abnormal cell-level processes. These techniques are based on multicomponent relaxometry or diffusion, magnetization transfer, high-dimensional imaging or spectroscopy, susceptibility imaging, cerebral functioning, etc. However, their integration in clinical trials and investigations is still limited. This session will provide an overview about these emerging MRI/MRS techniques, their value and application in studying aging. The overarching goal is to generate further interest within the scientific community, disseminate and integrate these mature and unique noninvasive approaches in research settings and routine clinical protocols to advance our understanding of aging processes.

